# Metabolic Syndrome and Chronic Kidney Disease in an Adult Korean Population: Results from the Korean National Health Screening

**DOI:** 10.1371/journal.pone.0093795

**Published:** 2014-05-07

**Authors:** Yong Un Kang, Ha Yeon Kim, Joon Seok Choi, Chang Seong Kim, Eun Hui Bae, Seong Kwon Ma, Soo Wan Kim

**Affiliations:** Department of Internal Medicine, Chonnam National University Medical School, Gwangju, Korea; University of Verona, Ospedale Civile Maggiore, Italy

## Abstract

**Background:**

This study was aimed to examine the prevalence of metabolic syndrome (MS) and chronic kidney disease (CKD), and the association between MS and its components with CKD in Korea.

**Methods:**

We excluded diabetes to appreciate the real impact of MS and performed a cross-sectional study using the general health screening data of 10,253,085 (48.86±13.83 years, men 56.18%) participants (age, ≥20 years) from the Korean National Health Screening 2011. CKD was defined as dipstick proteinuria ≥1 or an estimated glomerular filtration rate (eGFR) <60 ml/min/1.73 m^2^.

**Results:**

The prevalence of CKD was 6.15% (men, 5.37%; women, 7.15%). Further, 22.25% study population had MS (abdominal obesity, 27.98%; hypertriglyceridemia, 30.09%; low high-density cholesterol levels, 19.74%; high blood pressure, 43.45%; and high fasting glucose levels, 30.44%). Multivariate-adjusted analysis indicated that proteinuria risk increased in participants with MS (odds ratio [OR] 1.884, 95% confidence interval [CI] 1.867–1.902, *P*<0.001). The presence of MS was associated with eGFR<60 mL/min/1.73 m^2^ (OR 1.364, 95% CI 1.355–1.373, *P*<0.001). MS individual components were also associated with an increased CKD risk. The strength of association between MS and the development of CKD increase as the number of components increased from 1 to 5. In sub-analysis by men and women, MS and its each components were a significant determinant for CKD.

**Conclusions:**

MS and its individual components can predict the risk of prevalent CKD for men and women.

## Introduction

Metabolic syndrome (MS) includes various metabolic abnormalities that have been associated with cardiovascular disease, stroke, and all-cause mortality in the general population [1]. The components of MS include central obesity, elevated blood pressure, and impaired fasting glucose and high triglyceride (TG) and low high-density lipoprotein (HDL) cholesterol levels, and these components are present in approximately 20% adults in USA [2]. In Korea, recent changes in lifestyle and diet have resulted in an increased prevalence of MS, becoming an important public health concern. The prevalence of MS has been reported to be about 14.2% in men and 17.7% in women in the general population [3]. Another study suggested that the prevalence of MS has increased to approximately 19.0% [4].

The burden of chronic kidney disease (CKD) has also been increasing worldwide over the last decade and is expected to increase further [5]. The increased incidence of CKD in recent years paralleled with an increasing prevalence of MS [6], [7]. Previous observational studies have reported an independent association between MS and microalbuminuria or proteinuria and CKD [8], [9]. In a large cohort survey, both MS and microalbuminuria had strong adverse effects on the estimated glomerular filtration rate (eGFR), and this relationship was even more pronounced in the presence of both factors [10]. However, a few studies have shown statistically insignificant association between MS and low eGFR after adjustment for albuminuria [11].

Additionally, the reported studies used varying definitions for MS and studied different populations. Thus, the data on MS or its relationship with CKD in large population-based studies are limited. Therefore, we aimed to assess the prevalence of MS and CKD, and the association between MS and its components with CKD in an adult Korean population.

## Materials and Methods

### Study Population

Korean National Health Screening is an annual health examination to improve the health of people and reduce health-care costs by preventing cardiocerebrovascular diseases affected by lifestyle habits, such as hypertension, diabetes, dyslipidemia, and by detecting five major forms of cancer at an early stage. General Health Screening was the first-step screening test for an early detection. We performed a cross-sectional study using the general health screening data of 11,828,803 participants (age≥20 years) from the 2011 Korean National Health Screening database. Of these, 10,549,230 subjects with information on renal function, such as serum creatinine and urine analysis, and all parameters related to MS were included in this analysis. 296,145 subjects with diabetes were excluded to appreciate the real impact of MS. Finally, 10,253,085 subjects were recruited in our study to assess the prevalence and association between MS and CKD.

This study was approved by the institutional review board of Chonnam National University Hospital, Gwangju, Republic of Korea. This institutional review board waived the need for consent given the retrospective design of the project. The study was performed in accordance with the Helsinki Declaration of 1975, as revised in 2000.

### Data Collection

Health examination included data for anthropometric measurement, blood pressure, and blood chemistry tests. Information on sex, age, and other pertinent medical data were obtained. Anthropometric measurements, including height, weight, and waist circumference, were determined, and the body mass index (BMI) was calculated by dividing the weight in kilograms by height in meters squared. Trained nurses measured blood pressure at the health screening facilities using a standard protocol. Blood samples were collected in the morning after an overnight fast of at least 8 h. Plasma glucose levels were measured using a hexokinase enzyme reference method. At the health screening facilities, serum HDL cholesterol and TG levels were measured enzymatically by using a commercially available reagent mixture, and serum creatinine was measured using the modified Jaffe kinetic reaction.

### Definitions

MS was defined using the current criteria in the National Cholesterol Education Program Adult Treatment Panel III (ATP III) (NCEP) guidelines with a modification for waist circumference. Specifically, elevated blood pressure was defined as systolic or diastolic blood pressure of 130/85 mmHg or higher and the current use of antihypertensive medication. Elevated blood glucose level was defined as a fasting blood glucose level of ≥100 mg/dL or use of glucose level-lowering medicine. Low HDL cholesterol level was defined as that *<*40 mg/dL in men and *<*50 mg/dL in women. Hypertriglyceridemia was defined as a serum TG level of ≥150 mg/dL. Abdominal obesity was defined as a waist circumference *>*90 cm in men and *>*80 cm in women according to the Asia-Pacific criteria. MS was defined as the presence of three or more of these five components [12].

Diabetes mellitus (DM) was defined as a self-reported history of a previous diagnosis of diabetes or a fasting plasma glucose level of ≥126 mg/dL. Hypertension (HT) was defined as self-reported history of a previous diagnosis of HT or systolic blood pressure ≥140 mmHg or diastolic blood pressure ≥90 mmHg. eGFR was calculated using The Chronic Kidney Disease Epidemiology Collaboration (CKD-EPI) equation [13]. The definition of proteinuria was based on a dipstick urinalysis score of 1+ or more. CKD was defined as an eGFR*<*60 mL/min/1.73 m^2^ or dipstick proteinuria (≥1+) [14].

### Statistical Analysis

The prevalence of CKD, MS, and its individual components (abdominal obesity, high blood pressure, and high TG, low HDL cholesterol, and high fasting glucose levels) was determined for the overall population. Data were presented as the mean ± SD for continuous variables and as proportions for categorical variables. The characteristics of the subjects were compared using the chi-squared test for categorical variables. Student’s *t*-test was used for continuous variables. Multivariate logistic regression was used to estimate odds ratio (OR), and 95% confidence interval (CI) was used for determining the relationship between MS and proteinuria or eGFR*<*60 mL/min/1.73 m^2^. Covariates considered as potential confounders (age and sex) were included in multivariable models. Model 1 was unadjusted, model 2 included age and sex. For the eGFR analysis, model 3 included age, sex, and proteinuria. The associations between MS components and CKD were analysed using multivariate logistic regression models after adjustments for age and sex. Statistical analysis was performed with the SAS (Version 8.2, SAS Institute Inc., Cary, NC, USA). A *P*-value of <0.05 was considered statistically significant.

## Results

### Clinical Characteristics

We studied 10,253,085 (56.18% men) participants (mean age, 48.86±13.83 years). Women had significantly older age, higher proportion of hypertension, total cholesterol levels, low-density lipoprotein cholesterol levels, HDL cholesterol levels, and proportion of eGFR<60 mL/min/1.73 m^2^ but lower BMI, waist circumference, TG levels, systolic blood pressure, diastolic blood pressure, fasting glucose levels, eGFR, and serum creatinine levels than men. However, proportion of proteinuria was not different between men and women (Table 1).

**Table 1:** Clinical characteristics.

	Overall	Men	Women	*P*
Number of subjects	10,253,085	5,760,643 (56.18)	4,492,442 (43.82)	<0.001
Age (years)	46.86±13.83	45.12±13.40	49.10±14.05	<0.001
Hypertension (%)	238,989 (2.33)	123,447 (2.14)	115,542 (2.57)	<0.001
Body mass index (kg/m^2^)	23.88±3.23	24.28±3.07	23.46±3.36	<0.001
Waist circumference (cm)	80.67±9.02	83.80±7.83	76.66±8.84	<0.001
TC (mg/dL)	195.84±35.82	194.43±35.08	197.52±36.67	<0.001
LDL cholesterol (mg/dL)	114.74±33.02	113.41±32.48	116.45±33.62	<0.001
Triglyceride (mg/dL)	132.71±72.26	146.52±77.92	115.01±59.78	<0.001
HDL cholesterol (mg/dL)	54.66±13.45	51.99±12.62	58.07±13.70	<0.001
Systolic blood pressure (mmHg)	122.65±14.71	124.50±13.76	120.08±15.49	<0.001
Diastolic blood pressure (mmHg)	76.44±9.89	77.97±9.58	74.48±9.93	<0.001
Fasting glucose (mg/dL)	95.81±18.40	97.27±19.92	93.92±16.04	<0.001
eGFR (mL/min/1.73 m^2^)	90.02±19.64	91.19±19.24	90.81±20.14	<0.001
Serum creatinine (mg/dL)	0.96±0.71	1.06±0.75	0.82±0.62	<0.001
Proteinuria (%)	202,928 (1.98)	113,838 (1.98)	89,090 (1.98)	0.426
eGFR<60 mL/min/1.73 m^2^ (%)	459,032 (4.48)	213,207 (3.70)	245,825 (5.47)	<0.001
Chronic kidney disease (%)	630,420 (6.15)	309,360 (5.37)	321,060 (7.15)	<0.001
Metabolic syndrome (%)	2,281,675 (22.25)	1,270,105 (22.05)	1,011,570 (22.52)	<0.001
Individual component (%)				
Abdominal obesity	2,868,480 (27.98)	1,266,208 (21.98)	1,602,272 (35.67)	<0.001
Hypertriglyceridemia	3,085,216 (30.09)	2,163,829 (37.56)	921,387 (20.51)	<0.001
Low HDL cholesterol	2,023,489 (19.74)	786,802 (13.66)	1,236,687 (27.53)	<0.001
High blood pressure	4,454,799 (43.45)	2,757,750 (47.87)	1,697,049 (37.78)	<0.001
High fasting glucose	3,120,707 (30.44)	1,963,828 (34.09)	1,156,879 (25.75)	<0.001

Abbreviations: TC, total cholesterol; LDL, low-density lipoprotein; HDL, high-density lipoprotein; eGFR, estimated glomerular filtration rate.

### Prevalence of MS and CKD

The overall prevalence of CKD was 6.15% (men, 5.37%; women, 7.15%). The prevalence of CKD according to the stage was 0.81% in stage 1, 0.86% in stage 2, 3.52% in stage 3, and 0.96% in stage 4 or 5 (Table 2). We identified 2,281,675 (22.25%) subjects who met the current ATP III criteria. Among all subjects, 27.98% had abdominal obesity, 30.09% had hypertriglyceridemia, 19.74% had low HDL cholesterol levels, 43.45% had high blood pressure, and 30.44% had high fasting glucose levels. Women had significantly higher proportion of abdominal obesity and low HDL cholesterol but lower that of hypertriglyceridemia, high blood pressure, and high fasting glucose than men (Table 1).

**Table 2:** The prevalence of kidney function and chronic kidney disease stage.

Kidney function				Chronic kidney disease			
eGFR^a^	Overall^b^	Men^b^	Women^b^	Stage	Overall^b^	Men^b^	Women^b^
≥90	5,492,524 (53.57)	3,096,868 (53.76)	2,393,565 (53.33)	1	82,770 (0.81)	44,763 (0.78)	38,007 (0.85)
60–89	4,301,529 (41.95)	2,450,568 (42.54)	1,850,961 (41.20)	2	88,618 (0.86)	51,390 (0.89)	37,228 (0.83)
30–59	360,641 (3.52)	156,994 (2.73)	203,647 (4.53)	3	360,641 (3.52)	156,994 (2.73)	203,647 (4.53)
<30	98,391 (0.96)	56,213 (0.97)	42,178 (0.94)	4 or 5	98,391 (0.96)	56,213 (0.97)	42,178 (0.94)
Total	10,253,085 (100)	5,760,643 (100)	4,492,442 (100)	All	630,420 (6.15)	309,360 (5.37)	321,060 (7.15)

Abbreviations: eGFR, estimated glomerular filtration rate.

a eGFR, mL/min/1.73 m^2^ by CKD-PEI equation.

b All *P* value for trend<0.001.

**Figure 1:**
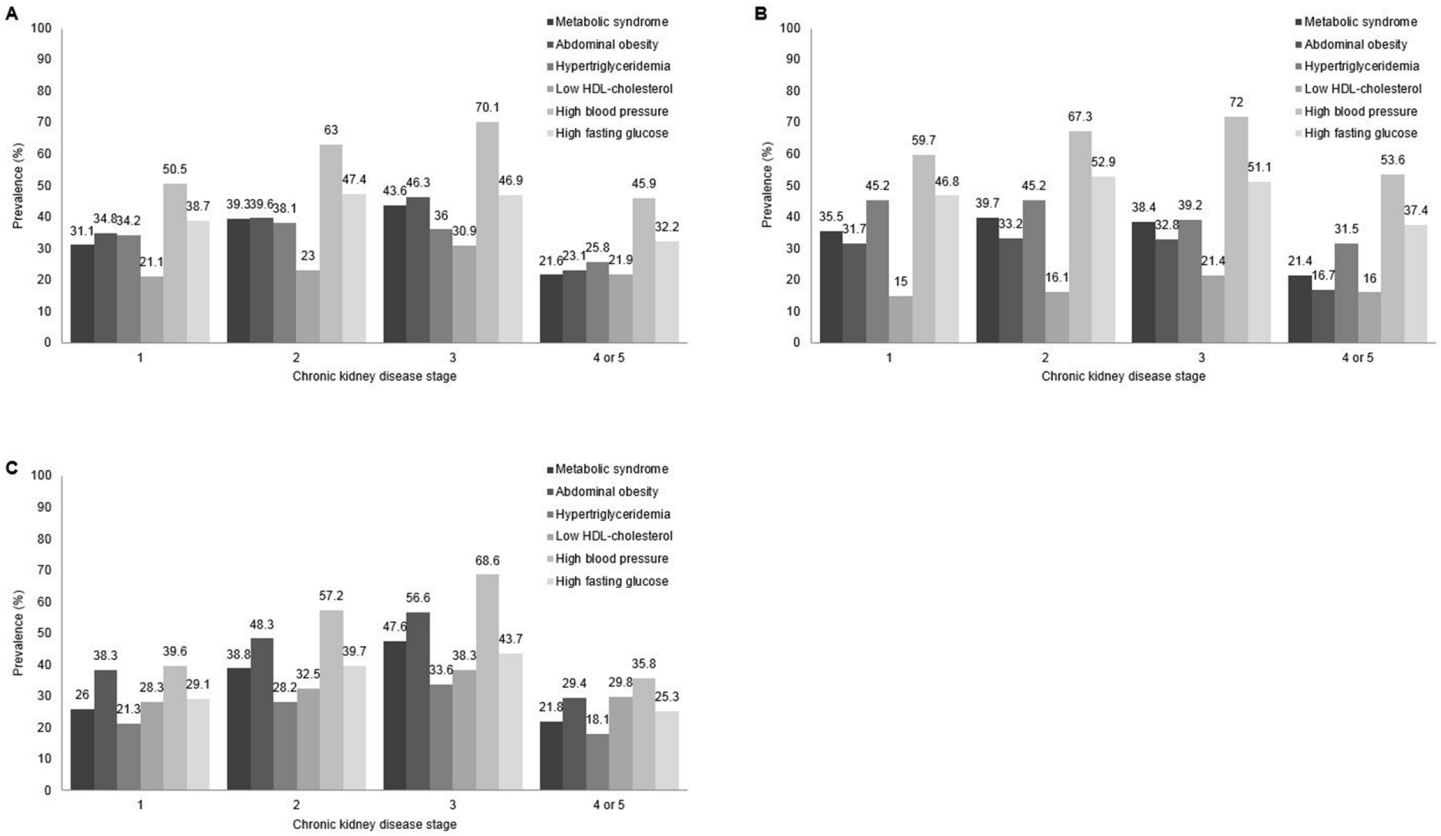
The prevalence of metabolic syndrome and its components according to chronic kidney disease stages. (A) Overall. (B) Men. (C) Women.

Subjects with MS had a higher prevalence of CKD than those without MS (10.48% vs. 4.91%, *P*<0.001). 37.93% of the participants with total CKD (n = 239,137) had MS (40.04% for abdominal obesity; 34.46% for hypertriglyceridemia; 27.1% for low HDL cholesterol; 62.74% for high blood pressure; and 43.61% for high fasting glucose). The prevalence of MS increased with an increase in CKD stage. On the other hand, that of MS in CKD stage 4 or 5 was the lowest compared to those with other CKD stages. High blood pressure was the most common MS component in each CKD stage (Figure 1).

### Association between MS and CKD

In multivariate logistic regression models, the participants with MS had a 1.884-fold increased OR (95% CI 1.867–1.902, *P*<0.001) for proteinuria compared with those without MS. In addition, the presence of MS was a significant determinant of eGFR<60 mL/min/1.73 m^2^ (OR 1.364, 95% CI 1.355–1.373, *P*<0.001) and CKD (OR 1.526, 95% CI 1.518–1.535, *P*<0.001) compared with those without MS. In sub-analysis by men and women, the presence of MS was also a significant determinant for proteinuria, eGFR<60 mL/min/1.73 m^2^, and CKD compared with those without MS (Table 3).

**Table 3:** Odds ratios for the association between metabolic syndrome and chronic kidney disease.

Description of model covariates	Odds ratio (95% CI)		
	Overall^a^	Men^a^	Women^a^
Proteinuria			
Model 1: unadjusted	2.148 (2.128–2.167)	2.317 (2.290–2.346)	1.947 (1.920–1.974)
Model 2: adjusted for age, sex	1.884 (1.867–1.902)	2.071 (2.046–2.096)	1.815 (1.788–1.843)
eGFR<60 mL/min/1.73 m^2^			
Model 1: unadjusted	2.328 (2.314–2.342)	1.866 (1.849–1.884)	2.806 (2.783–2.829)
Model 2: adjusted for age, sex	1.400 (1.391–1.409)	1.450 (1.437–1.464)	1.366 (1.353–1.378)
Model 3: adjust for age, sex, proteinuria	1.364 (1.355–1.373)	1.394 (1.381–1.407)	1.341 (1.329–1.353)
Chronic kidney disease			
Model 1: unadjusted	2.268 (2.256–2.280)	2.000 (1.984–2.015)	2.557 (2.538–2.576)
Model 2: adjusted for age, sex	1.526 (1.518–1.535)	1.626 (1.613–1.639)	1.452 (1.440–1.463)

Abbreviations: eGFR; estimated glomerular filtration rate; CI, confidence interval.

a All *P* value for trend<0.001.

### Association between MS Components and their Risk for Development of CKD

**Figure 2:**
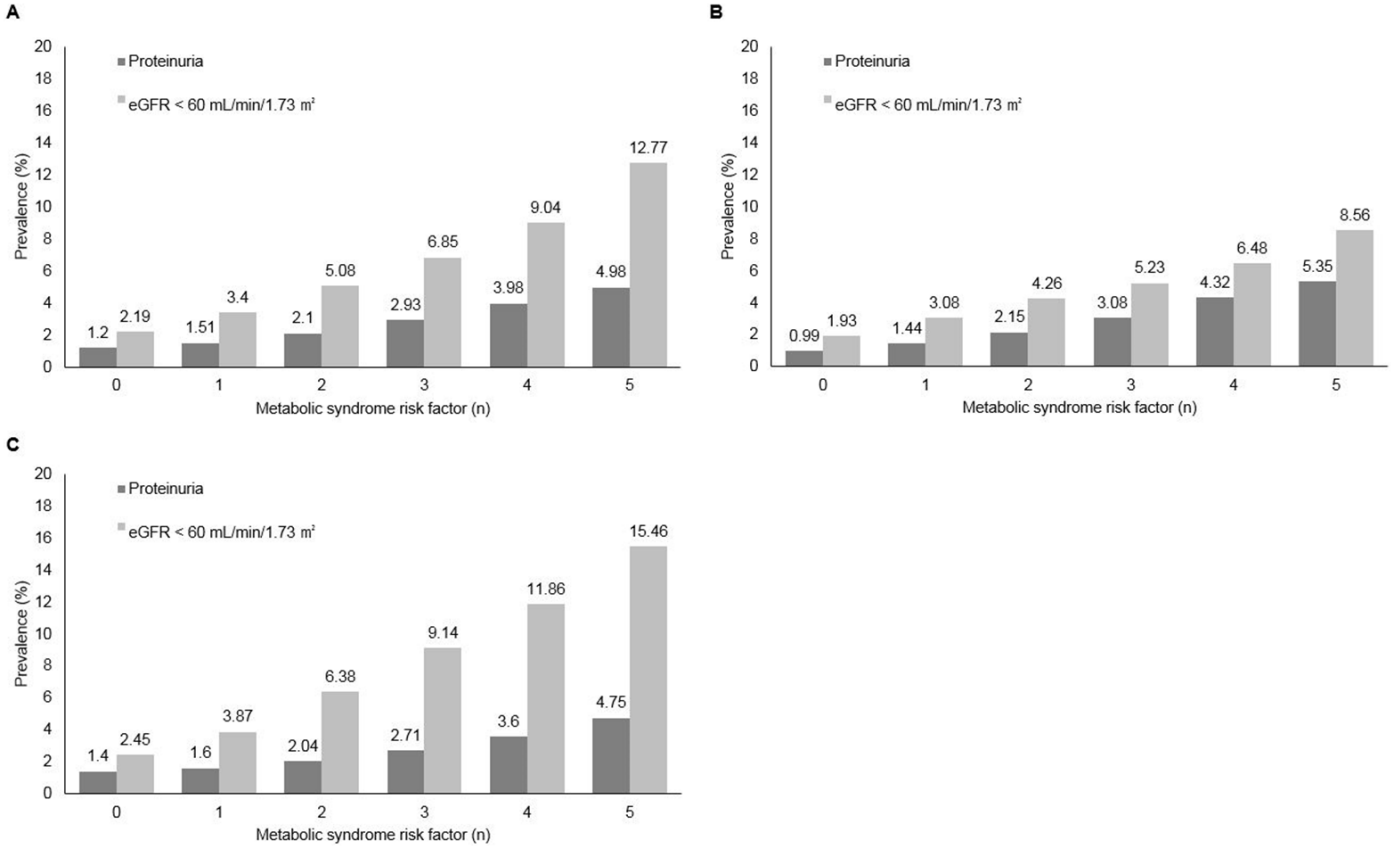
The prevalence of markers of chronic kidney disease according to number of metabolic syndrome components. (A) Overall. (B) Men. (C) Women.

In models adjusted for age and sex, we further examined the associations of individual components of MS and the risk of CKD by dividing each components of MS, in quartiles and making the groups accordingly. Increasing component levels of MS showed a positive association with the development of CKD compared with the lowest quartile for men and women, respectively. On the other hand, decreasing HDL cholesterol levels was linked clearly to CKD compared with the lowest quartile for men and women, respectively. The OR for CKD for high fasting glucose as an MS component was higher than that for other MS components (Table 4). The prevalence of markers of CKD was higher with increasing number of MS components (Figure 2). The participants with 1–5 component of MS had an increased OR for CKD: 1.062 (95% CI 1.053–1.071), 1.281 (95% CI 1.270–1.292), 1.575 (95% CI 1.561–1.590), 1.942 (95% CI 1.921–1.962), and 2.367 (95% CI 2.329–2.406) compared with those with no MS components (*P*<0.001). In sub-analysis for men and women, the strength of association between MS and the development of CKD also appeared to increase as the number of components increased from 1 to 5 (Table 4).

**Table 4:** Association between metabolic syndrome components and the risk of development of chronic kidney disease.

Variables	Odds ratio (95% CI)		
	Overall	Men	Women
Abdominal obesity^a^			
Q2	1.132 (1.121–1.142)^e^	1.261 (1.243–1.279)^e^	1.048 (1.036–1.061)^e^
Q3	1.228 (1.217–1.238)^e^	1.320 (1.303–1.338)^e^	1.153 (1.139–1.166)^e^
Q4	1.487 (1.475–1.500)^e^	1.691 (1.669–1.714)^e^	1.376 (1.361–1.391)^e^
Hypertriglyceridemia^a^			
Q2	1.140 (1.129–1.151)^e^	1.132 (1.118–1.146)^e^	1.159 (1.142–1.176)^c^
Q3	1.199 (1.188–1.210)^e^	1.204 (1.189–1.220)^e^	1.200 (1.183–1.217)^e^
Q4	1.308 (1.296–1.320)^e^	1.321 (1.305–1.338)^e^	1.302 (1.284–1.321)^e^
Low HDL cholesterol^a^			
Q2	0.878 (0.866–0.891)^e^	0.864 (0.843–0.886)^e^	0.879 (0.864–0.895)^d^
Q3	0.828 (0.816–0.840)^e^	0.799 (0.779–0.819)^e^	0.837 (0.822–0.852)^e^
Q4	0.734 (0.726–0.742)^e^	0.654 (0.642–0.665)^e^	0.778 (0.769–0.788)^e^
Systolic blood pressure^a^			
Q2	1.154 (1.144–1.163)^e^	1.233 (1.220–1.245)^e^	1.032 (1.017–1.047)^e^
Q3	1.201 (1.191–1.212)^e^	1.297 (1.280–1.313)^e^	1.119 (1.105–1.133)^d^
Q4	1.402 (1.390–1.414)^e^	1.555 (1.535–1.575)^e^	1.292 (1.278–1.307)^e^
Diastolic blood pressure^a^			
Q2	1.069 (1.056–1.082)^e^	1.111 (1.095–1.128)^e^	1.000 (0.981–1.021)^e^
Q3	1.163 (1.151–1.175)^e^	1.166 (1.150–1.183)^c^	1.162 (1.145–1.179)^e^
Q4	1.344 (1.330–1.358)^e^	1.388 (1.369–1.407)^e^	1.290 (1.270–1.311)^e^
High fasting glucose^a^			
Q2	1.137 (1.127–1.148)^e^	1.141 (1.127–1.156)^e^	1.133 (1.119–1.148)^e^
Q3	1.266 (1.255–1.277)^e^	1.256 (1.241–1.271)^e^	1.279 (1.263–1.296)^e^
Q4	1.568 (1.556–1.581)^e^	1.585 (1.568–1.602)^e^	1.549 (1.531–1.568)^e^
Number of components model^b^			
1 component	1.062 (1.053–1.071)^e^	1.198 (1.183–1.214)^e^	0.967 (0.955–0.979)^e^
2 components	1.281 (1.270–1.292)^e^	1.493 (1.474–1.512)^e^	1.125 (1.111–1.139)^e^
3 components	1.575 (1.561–1.590)^e^	1.875 (1.850–1.900)^e^	1.364 (1.346–1.382)^e^
4 components	1.942 (1.921–1.962)^e^	2.398 (2.360–2.436)^e^	1.653 (1.629–1.678)^e^
5 components	2.367 (2.329–2.406)^e^	2.911 (2.830–2.995)^e^	2.070 (2.027–2.112)^e^

Note: Adjusted for factors included in age and sex.

Abbreviations: CI, confidence interval; HDL, high-density lipoprotein.

a Reference group is lowest quartile (Q1) for each set of quartiles of factors.

b Reference group is 0 component for number of componants model.

c *P* value for trend>0.05.

d *P* value for trend<0.05.

e *P* value for trend<0.001.

## Discussion

The results of our study identified a strong, positive relationship between MS and the prevalence of CKD. A rise in the incidence of CKD in recent years has been associated with an increasing prevalence of MS [6], [7]. The prevalence of MS is about 24.7% of adults in USA [8]. A recent study demonstrated that the prevalence of MS has increased to approximately 19.0% in Korea [4]. Although we excluded subjects with diabetes to appreciate the real impact of MS, the present study reported an even higher prevalence of MS (22.25%), reflecting a rapidly increasing prevalence in Korea. The identification of MS is important in patients with a high risk of developing end-stage renal disease (ESRD).

Chen *et al*. [8] demonstrated a significant correlation between the number of MS traits and both albuminuria and eGFR<60 mL/min/1.73 m^2^. In a prospective cohort survey, Luk *et al*. [10] reported that the presence of MS independently predicted the development of CKD in subjects with type 2 diabetes. According to the National Health and Nutrition Examination survey, MS is independently associated with CKD in the general population and in non-diabetic adults [4], [15]. Palaniappan *et al.* [16] showed a higher risk for microalbuminuria in men and women with MS. These data support the importance of MS in the development of CKD. In a recent meta-analysis, although the results for proteinuria outcomes were debated because of the small number of prospective cohort studies, MS and its components were associated with the development of eGFR<60 mL/min/1.73 m^2^ and microalbuminuria or overt proteinuria [17]. All of these studies confirmed a relationship between MS and CKD. Also, our study results showed a strong relationship between MS and CKD in non-diabetic adult Korean population. In sub-analysis by men and women, MS was a significant determinant for CKD. These findings have important clinical and public health implications because the prevalence of the MS is rapidly increasing in Korea.

Our findings revealed each component of MS was an independent predictor of CKD. We analysed individual components to explore their differential effect in the presence of MS, and thus, the risk estimates were interpreted in the context of MS. In addition, the number of MS components and the risk for CKD showed a graded association. Moreover, the prevalence of markers of CKD increased with an increasing number of MS components. Therefore, therapeutic strategies that targeted individual MS components seem highly reasonable for preventing CKD.

Obesity is a significant risk factor for CKD. Visceral obesity is highly correlated with insulin resistance, and indices of visceral obesity may be more sensitive predictors of kidney disease than BMI [18]. Adipose tissue is a significant source of inflammatory and immunomodulatory factors; the interaction between adipocytes and macrophages may contribute to insulin resistance and many of the features that characterise MS [19]–[21]. Obesity occurs probably via the mechanisms associated with renal hyperfiltration and hyperperfusion as focal glomerulosclerosis and other histological changes have been observed in kidneys of obese patients [22], [23]. Previous studies reporting obesity as a significant risk factor for eGFR<60 ml/min/1.73 m^2^ used waist circumference criteria (a better measure of central adiposity) rather than BMI [24], [25]. Our study results indicated a 69.1% and 37.6% higher risk of developing CKD with abdominal obesity compared with the lowest quartile for men and women, respectively.

Dyslipidemia is also an important risk factor for proteinuria and decline of renal function [26]. In a prospective study of 12,728 subjects, high TG and low HDL cholesterol levels predicted an increased risk of renal dysfunction [27]. According to Ryu *et al*. [28] both high TG and low HDL cholesterol levels were associated with a significantly increased risk of CKD. These results remained unchanged, even after additional adjustment for incident HT and DM. The mechanisms underlying the contribution of lipids to renal injury are not completely understood. Dyslipidemia is associated with glomerular capillary endothelial and mesangial cell as well as podocyte injury, which further leads to mesangial sclerosis [29], [30]. The accumulation of lipoproteins in the glomerular mesangium can stimulate matrix production and glomerulosclerosis [31]. Although hypertriglyceridemia and low HDL cholesterol levels have been previously associated with an increased risk for CKD [26]–[28], these factors are often overlooked in clinical practice. Our results suggest that these could be potential targets for reducing the risk of CKD.

DM and HT are the most common risk factors for ESRD [14], and MS is frequently associated with these factors. The PAMELA (Pressioni Arteriose Monitorate E Loro Associazioni) population study revealed high normal blood pressure values and HT in 80% individuals with MS [32], [33]. According to the results of a previous study, insulin resistance and central obesity have been considered the main factors involved in hypertension pathophysiology associated with MS [34]. Recent community-based prospective observational cohort studies reported a significant risk for impaired fasting glucose and development of eGFR<60 mL/min/1.73 m^2^ in Asia [35],[36]. Evidence has shown that hyperinsulinemia occurs well before the onset of glucose intolerance, and this evidence suggests insulin resistance at levels of the muscle and liver, which is not directly indicated in the NCEP-ATP III criteria for MS. Thus, insulin resistance and subsequent hyperinsulinemia in addition to mild hyperglycemia may be accountable for the association between impaired fasting glucose level and CKD [37],[38]. In our analysis, we excluded subjects with diabetes to appreciate the real impact of MS. both high blood pressure and high fasting glucose levels were associated with significantly increased risk of CKD for men and women.

The present study has several limitations. First, the cross-sectional study design makes it difficult to infer causality between MS and the risk of developing CKD. Second, laboratory tests, specifically estimation of serum creatinine levels were performed in each hospital. Therefore, inter-laboratory variability may be present. A systematic difference in creatinine measurements between laboratories could have affected the association between MS and CKD. Third, CKD was defined as an eGFR*<*60 mL/min/1.73 m^2^ or dipstick proteinuria (≥1+). This definition didn’t include another kidney damages (e.g. abnormal urine sediment, abnormal blood and urine chemistry, abnormal imaging studies) and repeated measurements of creatinine levels and proteinuria were not performed. These factors could have led to underestimation or overestimation of the prevalence of CKD. Finally, other parameters associated with obesity and glucose metabolism, such as physical activity and alcohol consumption, were not evaluated in the present study. However, we believe most of these potential limitations should be consistently attenuated by the large sample size of the study.

In conclusion, our study confirmed MS and its individual components as a strong and independent risk factor for men and women with CKD. In addition, we found a graded relationship between the number of MS components and the risk for CKD. Our results emphasise that individuals with metabolic risk factors should be identified that an early stage and should undergo multidisciplinary interventions, particularly lifestyle modifications, to impede the development of CKD.
